# Physical, Physiological, Technical and Tactical Responses According to the Playing Position in Male Basketball: A Systematic Scoping Review

**DOI:** 10.5114/jhk/203326

**Published:** 2025-04-30

**Authors:** Diogo V. Martinho, Filipe Manuel Clemente, Miguel Ángel-Gomez, André Rebelo, Adam Field, Catarina C. Santos, Élvio R. Gouveia, José Afonso, Hugo Sarmento

**Affiliations:** 1 Faculty of Sport Sciences and Physical Education, University of Coimbra, Coimbra, Portugal.; 2 Laboratory of Robotics and Engineering Systems (LARSYS), Interactive Technologies Institute (ITI), Funchal, Portugal.; 3 School of Sport and Leisure, Polytechnic Institute of Viana do Castelo, Viana do Castelo, Portugal.; 4 Sport Physical Activity and Health Research & Innovation Center, Viana do Castelo, Portugal.; 5 Department of Biomechanics and Sport Engineering, Gdansk University of Physical Education and Sport, Gdansk, Poland.; 6 Faculty of Physical Activity and Sport Sciences, Polytechnic University of Madrid, Madrid, Spain.; 7 CIDEFES, Research Center in Sport, Physical Education, and Exercise and Health, Lusófona University, Lisbon, Portugal.; 8 COD, Center of Sports Optimization, Sporting Clube de Portugal, Lisbon, Portugal.; 9 Department of Sport and Exercise Science, Manchester Metropolitan University, Manchester, United Kingdom.; 10 Centre of Research, Education, Innovation, and Intervention in Sport (CIFI2D), Faculty of Sport, University of Porto, Portugal.; 11 Coimbra Education School, Polytechnic Institute of Coimbra, Coimbra, Portugal.; 12 Department of Physical Education and Sport, University of Madeira, Funchal, Portugal.; 13 CIPER, FCDEFUC, University of Coimbra, Coimbra, Portugal.

**Keywords:** match analysis, basketball demands, internal load, external load, performance

## Abstract

Understanding how playing position influences physical, physiological, technical, and tactical demands in basketball is essential for optimizing training. Despite numerous studies examining these demands, there remains a need for a comprehensive review focused specifically on male basketball players. This scoping review aimed to summarize these demands during training sessions and games among male professional and semiprofessional basketball players according to playing positions. Following the PRISMA guidelines and its extensions for scoping reviews, four databases (PubMed, Scopus, SPORTDiscus, and Web of Science) were searched. Studies were included if they featured male professional or semiprofessional basketball players, assessments during training or games, and reported relevant demands. Forty-seven manuscripts were reviewed. Key findings revealed notable differences between positions: (i) (i) guards covered greater distances, performing more accelerations and decelerations compared to forwards and centers; (ii) forwards engaged in more high-speed and high-intensity running; (iii) centers demonstrated higher averages in successful shots and rebounds; and (iv) physiological responses, particularly heart rate, were predominantly higher among centers. In conclusion, this review provides coaches with critical insights into position-specific physical and physiological demands in basketball. Notably, methodological inconsistencies across the studies reviewed were observed. Hence, establishing standardized assessment methodologies and creating a common framework for normalizing physical, physiological, technical, and tactical variables is crucial for enhancing research comparability and practical application.

## Introduction

In basketball, assessing physical and physiological variables is central to understanding training responses and adaptations, examining fatigue levels, and potentiating recovery strategies (Bourdon et al., 2017; Gabbett and Whiteley, 2017; Halson, 2014). In addition, the technical and tactical aspects of the game provide vital information for coaches to design practices and recruit players (Garcia et al., 2013; Mateus et al., 2020). The characterization of the physical demands of basketball is challenging. Although time-motion analysis is the most reported approach to assess players’ activities (Abdelkrim et al., 2007; Conte et al., 2015; Torres-Ronda et al., 2016), data interpretation depends on a specialist who is vulnerable to errors, and requires software and time (Fox et al., 2017). Consequently, microtechnology devices (global and local positioning systems and inertial movement units (IMUs) have been used to describe the physical demands of professional basketball players (García et al., 2021; Portes et al., 2020; Salazar et al., 2020). The physiological demands imposed on players during the competition and training sessions have been investigated via heart rate monitors (Abdelkrim et al., 2007; Torres-Ronda et al., 2016), blood lactate concentration (Ben Abdelkrim et al., 2010; Narazaki et al., 2009) and rate of perceived exertion scales (Conte et al., 2018; Manzi et al., 2010). The number of published research articles on physical, physiological, technical, and tactical variables in basketball has increased significantly in recent years (Fox et al., 2020; Garcia et al., 2021; Gomez et al., 2017), however, there is uncertainty as to whether, and how, these demands differ between positional groups.

Two reviews have describe the physical and physiological demands of basketball in female athletes (Espasa-Labrador et al., 2023) and considered variations at the competitive level (Petway et al., 2020); however, they did not examine the impact of the playing position on basketball demands, which may lead practitioners to generalize training prescriptions. Another two reviews summarized the physical and physiological demands experienced by players relative to playing positions in male (Stojanović et al., 2018) and female basketball players (Power et al., 2022). On the one hand, these reviews provide insights into the physical and physiological demands during training and games; on the other hand, the findings in females should not be generalized to males. In addition, data from reviews that included male players (Stojanovic et al., 2018) were limited to frequencies, distances, and duration obtained from time-motion analyses. Given the limitations of time-motion analyses in interpreting the physical demands in basketball (Fox et al., 2017), and the current use of microtechnology to examine the physical demands (Pérez-Chao et al., 2023), another review is needed. Furthermore, previous studies ignored the variation in the technical and tactical performance of positional groups.

Despite the significant advances in understanding the physical and physiological demands of basketball, there are still important areas that require further exploration. The available research has provided valuable insights into the physical demands of basketball, particularly when time-motion analyses and microtechnologies are used. However, much of this research has focused primarily on male athletes and physical variables, often overlooking the positional differences in key aspects of basketball performance. While microtechnology has enhanced our ability to assess player movements, its potential to shed light on how physical demands are linked to technical and tactical performances across different playing positions has yet to be fully analysed. Additionally, the variation in technical and tactical demands by playing position has been underexplored, leaving a gap in the understanding of how these factors interact with physical and physiological requirements. Addressing these gaps is crucial for developing more tailored, position-specific training and recovery strategies, emphasizing the need for further research that integrates all dimensions of performance.

Therefore, the aims of the present systematic scoping review were (1) to examine the impact of the playing position on physical, physiological, technical, and tactical demands in adult male professional or semiprofessional players, (2) to contextualize the methodologies and approaches used to explain activity profiles in training and competition, and (3) to identify literature gaps and provide suggestions for further research.

## Methods

This scoping review was developed according to the Cochrane instructions (Higgins et al., 2019) and followed two statements: the PRISMA 2020 guidelines (Page et al., 2021) and the respective extension for scoping reviews (Tricco et al., 2018). The protocol was registered on the Open Science Framework at doi.org/10.17605/OSF.IO/XEC6D.

### Eligibility Criteria

Published original studies and those available ahead-of-print in English, Portuguese or Spanish, were considered for the review without date restrictions. The inclusion criteria were as follows: 1) male professional or semiprofessional basketball players classified from Tier 3 (i.e., highly trained/national level) to Tier 5 (i.e., World Class) according to the Participation Classification Framework (McKay et al., 2022). Tier 4 specifically refers to basketball athletes competing at the elite/international level (McKay et al., 2022). These tiers were chosen to minimise any potential confounding factors in the conclusions of this review, particularly with respect to the training level; 2) the exposure needed to be assessed in a training or a game context; 3) studies that examined physical outcomes (e.g., distance covered, intensity thresholds, accelerations, decelerations, activity profile), physiological demands (e.g., heart rate, rate of perceived exertion) or technical/tactical performance (e.g., shooting percentage, assistance, rebounds); and 4) no restrictions were applied to the study designs eligible for inclusion.

### Information Sources and Source Strategy

Four electronic databases were searched: PubMed, Scopus, SPORTDiscus, and Web of Science (all databases) on the 4^th^ of August, 2024. The following search strategy was used: ((basket*) AND (train* OR match* OR game* OR competition* OR “match-play” OR “notational analysis” OR statistics) AND (“time-motion” OR demand* OR run* OR locomotor OR technic* OR perform* OR physical OR physiologic* OR “heart rate” OR distance OR intensity* OR “rate of perceived exertion” OR RPE OR lactate) AND (position* OR formation*)). The first author consulted the reference lists of the studies included in the present review to determine whether additional manuscripts should be added to the final list.

### Selection Process

Specialized reference manager software (EndNoteTM 21.0, ClarivateTM) was used to combine all the references. Then, duplicates were automatically removed and manually confirmed by two authors (D.V.M. and A.R.). The screening process was initially performed according to the title and abstract, and subsequently, the full texts of the papers were consulted to confirm that the studies met the inclusion criteria. Two independent authors (D.M.V. and A.R.) completed the screening process, and in the event of disagreement, a third author (H.S.) was contacted.

### Data Extraction and Data Items

The first authors developed a template to organize the relevant information. An Excel^®^ file was organized into three sheets: (1) physical data, (2) physiological outcomes, and (3) technical and tactical game data. The information about the sample size, the competitive level, the country, the number of teams analysed, classification for the playing position, qualitative and quantitative information about the output examined (physical, physiological, tactical or technical), was extracted by two authors (D.V.M. and A.R.).

For the physical variables, the information extracted considered, for example, total distance covered or distance covered at different intensity thresholds, accelerations, and decelerations. The mean and standard deviations, when reported, were collected as absolute or relative values (e.g., expressed per playing time, percentage of playing, or live time). Information about the methodologies (i.e., microtechnology or TMA) and instruments (i.e., model, brand, and sampling rate) used to obtain physical data was also included in the file. The mean and standard deviation of the physiological variables were retained for the analysis. In studies about technical and tactical variables, the following variables were extracted from each manuscript: data quality, and offensive and defensive variables. The corresponding authors were contacted when relevant data were not reported. When the data were presented graphically, specific software was used (GetData Graph Digitizer; http://www.getdata-graph-digitizer.com).

## Results

### Study Identification and Selection

**Figure 1:**
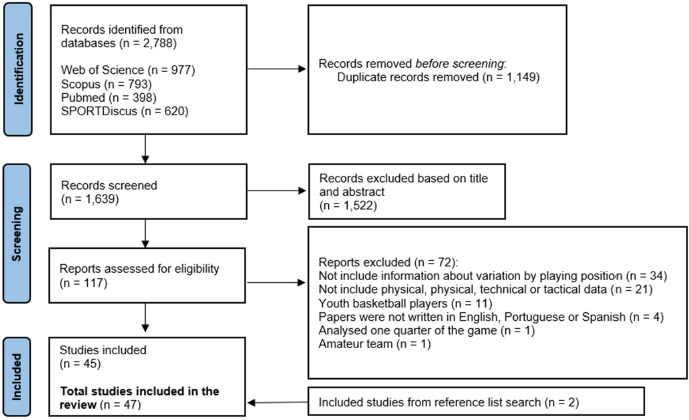
Flowchart of the review process.

The initial search of the four databases identified 2,788 manuscripts. Duplicates were removed (1,149 records), and 1,639 studies were screened by the title and the abstract. Of these, 1,522 manuscripts were omitted, and 117 records were consulted by full text, 72 of which were removed for the following reasons: studies did not include information about variation by playing position (n = 34); information about the physical, physiological, technical or tactical demands was not presented (n = 21); studies with youth players (n = 11); manuscripts were not written in English, Portuguese or Spanish (n = 4); the competitive level of the team analysed was not professional or semiprofessional (n = 1); and one study examined only one quarter of the game. Forty-five full texts were included in the present review. Two additional studies were identified as eligible during manual searches of reference lists. Finally, forty-seven full texts were included in this review (Figure 1).

### Characteristics of Studies

**Figure 2:**
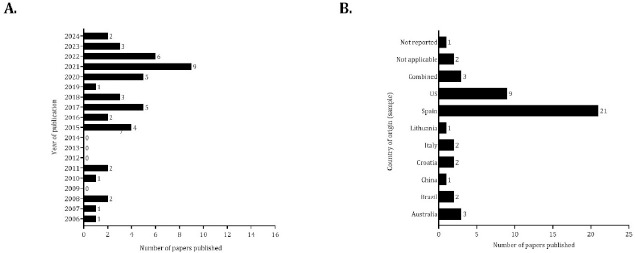
Number of studies published by year (panel A) and according to the country of the origin of the sample (panel B). Note: On panel B two studies combined data from Euroleague and Spanish Professional Basketball league (Vazquez-Guerrero and Garcia, 2020; Svilar et al., 2018), two studies (Escalante et al., 2010; Sampaio et al., 2008) used data exclusively from Euroleague or the European Basketball Championship and were classified as “not applicable”, one study did not report the country (Salazar et al., 2020)

**Figure 3:**
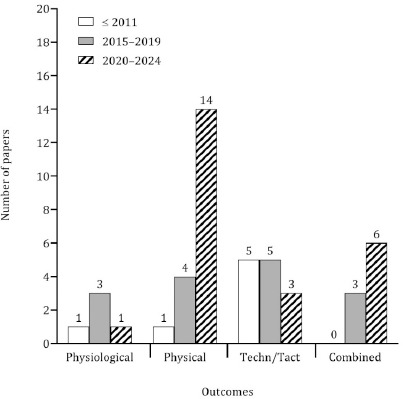
Number of papers (grouped in 5-year periods) about physiological, physical and tactical/technical variables. Tech/Tact: technical/tactical

**Figure 4:**
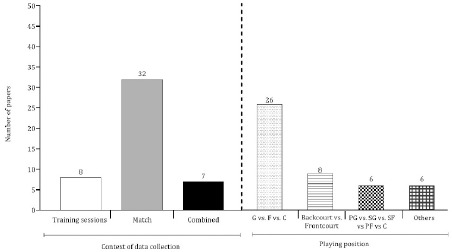
Number of papers considering the context of data collection and the classification by the playing position. Note: “Combined” refers to studies that examined the outcomes in training and competitions. The classification of the playing position considered the three most frequent categories found in literature. The remaining categories were classified as “others”. G: guards; F: forwards; C: centers; PG: point guards; SG: shooting guards; SF: small forwards; PF: power forwards

Tables 1 and 2 describe the characteristics of each study included in the present review, and Figures 2–4 summarize the main information extracted from the tables (Bordon et al., 2021; Courel-Ibáñez et al., 2017; Daniel et al., 2016, 2017; Dehesa et al., 2015; Escalante et al., 2010; Escudero-Tena et al., 2021; Ferioli et al., 2020; Gamonales et al., 2023; García et al., 2020, 2022a, 2022b, 2022c, 2022d; Gervasi et al., 2023; Gómez et al., 2018; Heishman et al., 2020; Ibáñez et al., 2024; López et al., 2021; Lorenzo Calvo et al., 2017; Madinabeitia et al., 2023; Mateus et al., 2015; Page et al., 2007; Pernigoni et al., 2021; Puente et al., 2017; Russell et al., 2021b; Sampaio et al., 2006, 2008; Sansone et al., 2021; Saucier et al., 2021; Scanlan et al., 2011, 2015; Sindik and Jukić, 2011; Sindik, 2015; Stone et al., 2022; Svilar et al., 2018; Trapero et al., 2019; Vaquera et al., 2008; Vázquez-Guerrero et al., 2018, 2020; Vázquez-Guerrero and Garcia, 2021; Wang and Zheng, 2022; Williams et al., 2021; Yang, 2024; Zhang et al., 2017). Investigation into the physical, physiological, technical and tactical demands according to the playing position increased in 2015 (approximately 87% of the papers included in the review were published between 2015 and 2024). Seven papers were published from 2006 to 2011; however, the data were not found between 2012 and 2014 (Figure 2, Panel A). Figure 2 (Panel B) shows the number of studies published considering the country of origin of the sample analysed. The topic has received considerable attention in Spain and the U.S. (approximately 45% and 19% of studies were developed with professional or semiprofessional players from Spain or America, respectively). More than 50% of the papers included only one team, and 15 studies (approximately 33%) did not report the number of teams analysed.

**Table 1:** Characteristics of studies included in the present review.

Study	Country	Competition	Outcome examined	Context of data collection (N)	Classification by playing position (N)	Teams analysed (N)
Yang (2024)	China	State Chinese Basketball League	Physical, physiological	Match (n = 18)	Guards, forwards, centers	1
Ibáñez et al. (2024)	Spain	Spanish Professional Basketball League	Physical, physiological	Training session (n = 9)	Guards (n = 3), forwards (n = 5), centers (n= 4)	1
Madinabeitia et al. (2023)	Spain	Spanish Professional Basketball League	Technical	Match (n = 335)	Point guards, shooting guards, shooting forwards, point forwards, centers	NR
Gervasi et al. (2023)	Italy	Italian Professional Basketball League	Physical	Match (n = 15)	Point guards (n = 2), guards (n = 4), forwards (n = 5), centers (n = 2)	1
Gamonales et al. (2023)	Spain	Spanish Professional Basketball League	Physical	Training session	Point guards (n = 5), shooting guards (n = 2), small forwards (n = 4), power forwards (n = 1), centers (n = 3)	1
Wang and Zheng (2022)	US	National Basketball Association	Technical	Match	Point guards (n = 48, shooting guards (n = 59), small forwards (n = 54), power forwards (n = 54), centers (n = 54)	NR
Stone et al. (2022)	US	NCAA Division I	Physical	Match (n = 27)	Guards (n = 4), forwards (n = 3), centers (n = 4)	NR
Garcia et al. (2022d)	Spain	Second Division Spanish Basketball League	Physical	Match (n = 17), training session	Guards (n = 7), forwards (n = 3), centers (n = 3)	1
Garcia et al. (2022c)	Spain	Second Division Spanish Basketball League	Physical	Match (n = 11)	Guards, forwards, centers	1
Garcia et al. (2022b)	Spain	Third Division Spanish Basketball League	Physical, physiological, technical	Match (n = 6)	Backcourt (n = 8), frontcourt (n = 6)	1
Garcia et al. (2022a)	Spain	Second Division Spanish Basketball League	Physical	Match (n = 12)	Backcourt (n = 5), frontcourt (n = 7)	1
Williams et al. (2021)	Australia	Queensland Basketball League	Physical, physiological	Match (n = 18), training session	Backcourt (n = 4), frontcourt (n = 4)	1
Vázquez-Guerrero and Garcia (2021)	-	Spanish Professional Basketball League, Euroleague	Physical	Match (n = 1)	Guards (n = 11), forwards (n = 5), centers (n = 5)	2
Saucier et al. (2021)	US	NCAA First Division	Physical	Match (n = 35), training session (n = 77)	Guards (n = 7), forwards (n = 4), centers (n = 4)	1
Sansone et al. (2021)	Spain	Semi-professional level^1^	Physiological, technical	Match, training session	Guards (n = 5), forwards (n = 6), centers (n = 3)	1
Russel et al. (2021b)	US	National Basketball Association	Physical	Match, training session	Backcourt, frontcourt	1
Pernigoni et al. (2021)	Lithuania	Third Division Lithuanian Basketball League	Physical	Match (n = 3)	Backcourt (n = 6), frontcourt (n = 5)	1
López et al. (2021)	Spain	Second Division Spanish Basketball League	Physiological	Training session	Point guards (n =2), perimeters (n = 4), inside (n = 4)	1
Escudero-Tena et al. (2021)	Spain	Spanish Professional Basketball League	Technical	Match (n = 327)	Point guards, shooting guards, forwards, power forwards, centers	NR
Bordon et al. (2021)	Spain	Second Division Spanish Basketball League	Physical, physiological	Training session	Inside, outside	1
Heishman et al. (2020)	US	NCAA First Division	Physical	Training session (n = 22)	Guards (n = 7), forwards and centers (n = 7)	NR

NR (not reported)

**Table 2:** Characteristics of studies included in the present review.

Study	Country	Competition	Outcome examined	Context of data collection (N)	Classification by playing position (N)	Teams analysed (N)
Vázquez-Guerrero et al. (2020)	Spain	Spanish Professional Basketball League, Euroleague	Technical, physical	Match (n = 63), training session (n = 315)	Point guards, shooting guards, small forwards, power forwards, centers	1
Salazar et al. (2020)	-	Elite level^1^	Physical	Match (n = 5)	Guards (n = 6), forwards (n = 4), centers (n = 7)	NR
Garcia et al. (2020)	Spain	Second Division Spanish Basketball League	Physical	Match (n = 17)	Guards (n = 7), forwards (n = 3), centers (n = 3)	1
Ferioli et al. (2020)	Italy	Italian Professional Basketball League, Second Division Italian Basketball League	Physical	Match (n = 10)	Guards (n = 22), forwards (n = 14), centers (n = 8)	6
Trapero et al. (2019)	Spain	Spanish Professional Basketball League, Spanish U18 team	Physical	Training session	Guards (U18: n = 5, SPBL: n = 5), forwards (U18: n = 5, SPBL: n = 4), centers (U18: n = 2, SPBL: n = 3)	2
Vázquez-Guerrero al. (2018)	Spain	Spanish Professional Basketball League	Physical	Match (n = 2)	Point guards (n = 4), shooting guards (n = 6), power forwards (n = 4), centers (n = 5)	1
Svilar et al. (2018)	-	Spanish Professional Basketball League, Euroleague	Physical, physiological	Training sessions (n = 300)	Guards (n = 4), forwards (n = 4), centers (n = 3)	1
Gomez et al. (2018)	Spain	Spanish Professional Basketball League	Technical	Match (n = 104)	Guards (n = 32), forwards (n = 32), centers (n = 8)	NR
Zhang et al. (2017)	US	National Basketball Association	Technical	Match (n = 699)	Guards (n = 59), forwards (n = 140), centers (n = 59)	NR
Puente et al. (2017)	Spain	Tournament (different competitive levels)	Physical, physiological	Match	Guards (n = 8), forwards (n = 8), centers (n = 9)	NR
Daniel et al. (2017)	Brazil	Brazil National League	Physiological	Match (n = 1)	Point guards, shooting guards, small forwards, power forwards, centers	NR
Courte-Ibáñez et al. (2017)	US	National Basketball Association	Technical, tactical	Match (n = 25)	Point guards, shooting guards, shooting forwards, power forwards, centers	NR
Calvo Lorenzo et al. (2017)	Spain	Spanish Professional Basketball League	Tactical	Match (n = 40)	Outside (n = 30), inside (n = 26)	NR
Torres Ronda et al. (2016)	Spain	Spanish Professional Basketball League	Physical	Match (n = 7), training session (n = 32)	Point guards (n = 3), wingers (n = 6), centers (n = 5)	1
Daniel et al. (2016)	Brazil	Brazil National League	Physiological	Match (n = 6)	Point guards (n = 2), shooting guards (n = 2), small forwards (n = 2), centers (n = 3)	1
Sindik (2015)	Croatia	A-1 Croatia Basketball League	Technical	Match (n = 16)	Guards (n = 47), forwards and centers (n = 27)	9
Scanlan et al. (2015)	Australia	Queensland Basketball League	Physical	Match (n = 3)	Backcourt (n = 5), frontcourt (n = 7)	1
Mateus et al. (2015)	US	National Basketball Association	Technical, physical	Match (n = 712)	Guards (n = 180), forwards (n = 174), centers (n = 120)	NR
Dehesa et al. (2015)	Spain	Second Division Spanish Basketball League	Physiological	Training session (n = 12)	Guards (n = 2), forwards (n = 5), centers (n = 4)	1
Scanlan et al. (2011)	Australia	Queensland Basketball League	Physical	Match (n = 2)	Backcourt (n = 5), frontcourt (n = 5)	NR
Sindik and Jukic (2011)	Croatia	A-1 Croatia Basketball League	Technical	Match (n = 16)	Point guards (n = 18), shooting guards (n = 29), small forwards (n = 10), power forwards and centers (n = 17)	9
Escalante et al. (2010)	-	European Basketball Championship	Technical	Match (n = 54)	Guards (n = 77), forwards (n = 69), centers (n = 46)	NR
Vaquera et al. (2008)	Spain	Spanish Professional Basketball League	Physiological	Match (n = 5)	Point guards (n = 2), forwards (n = 3), centers (n = 3)	1
Sampaio et al. (2008)	-	Euroleague	Technical	Match (n = 225)	Guards (n = 493), forwards (n = 485), centers (n = 233)	NR
Page et al. (2007)	US	National Basketball Association	Technical	Match	Point guards, small forwards, power forwards, centers	29
Sampaio et al. (2006)	US, Spain, Portugal	National Basketball Association, Spanish Professional Basketball League, Portuguese Professional League	Technical	Match (n = 12)	Guards (n = 75), forwards (n = 80), centers (n = 54)	NR

1 Competition was not reported. U18 (Under-18); SPBL (Spanish Professional Basketball League); NR (not reported)

The manuscripts were grouped into four different topics on the basis of the outcomes examined: physiological, physical, technical/tactical or combined. The latter group corresponded to manuscripts that covered more than one outcome (Figure 3). Between 2006 and 2010, examinations of physiological output in the context of training or competition were scarce (only three papers were found). The interest in technical and tactical characteristics has remained reasonably stable over the years, however, a substantial increase in the number of studies on the physical domain in the last five years has been noted. Figure 4 presents the context of data collection on the left side. More than 60% of the studies investigated physical, physiological, technical or tactical outcomes during the match, whereas only 15% of the manuscripts focused on training sessions. The right side of Figure 4 describes the classification used to group players according to the playing position. Twenty-six studies (approximately 55%) classified players as guards, forwards or centers, and a negligible percentage of studies adopted two (backcourt vs. frontcourt: 17%) or five categories (point guards, shooting guards, small forwards, power forwards or centers: 13%). Six studies used other terminologies to define playing positions (e.g., point guards, small forwards, power forwards, centers (Page et al., 2007); point guards, shooting guards, small forwards, centers (Daniel et al., 2016)).

### Methodological Characteristics

According to Fox et al. (2017), methods for measuring external demands can be classified into two major categories: time-motion analysis and microtechnology. For microtechnology devices, the brand and the sampling rate were also retrieved. Twenty-nine manuscripts presented data related to physical demands. In twenty-six studies, microtechnology devices and time-motion analysis were used. One study combined both methodological approaches, and two did not report the method used to examine the physical demands (Mateus et al., 2015; Zhang et al., 2017). A range of physical variables and thresholds were used across the studies, as shown in Tables 3 and 4. Fourteen studies presented total distance covered (Bordon et al., 2021; Gamonales et al., 2023; Garcia et al., 2020, 2022a, 2022b, 2022c, 2022d; Gervasi et al., 2023; Ibanez et al., 2024; Mateus et al., 2015; Puente et al., 2017; Saucier et al., 2021; Vazquez-Guerro and Garcia, 2021; Zhang et al., 2017), but only six studies related the distance covered per minute (Bondon et al., 2021; Gamonales et al., 2023; Garcia et al., 2022b; Ibanez et al., 2024; Puente et al., 2017; Vazquez-Guerreo et al., 2021; Zhang et al., 2017). Two studies focused on the most demanding scenarios, adjusting the distance covered for a specific period of time (Garcia et al., 2022c, 2022d). High-intensity running or high-speed running was reported in five studies relative to minutes of playing time (Ibanez et al., 2024; Gamonales et al., 2023; Garcia et al., 2022b; Puente et al., 2017; Vazquez-Guerrero and Garcia, 2022), although the thresholds widely varied across studies. Different terminologies have been applied to characterize accelerations and decelerations (e.g., maximal acceleration, intermediate acceleration, low acceleration, high-intensity acceleration, high-intensity deceleration, total acceleration, moderate acceleration, and moderate decelerations). Ten studies (Ibanez et al., 2024; Gamonales et al., 2023; Garcia et al., 2022a, 2022b, 2022c, 2022d; Puente et al., 2017; Saucier et al., 2021; Stone et al., 2022; Trapero et al., 2019) evaluated accelerations and decelerations and expressed both variables in different units (number, number^.^min^−1^ m^.^min^−1^). The thresholds used to define acceleration and deceleration were inconsistent in the previously mentioned studies.

**Table 3:** Methodological approaches of studies focused on physical performance.

Study	Methods used to measure external demands	Units	Variable	Threshold
Yang (2024)	Microtechnology (Catapult S7 device, 100 Hz)		Jumps LI	< 20 cm
			Jumps MI	20–40 cm
			Jumps HI	> 40 cm
			HI accelerations	-
			HI decelerations	-
			COD left	-
			COD right	-
		n·min^−1^	LI events	1.5–2.5 m·s^−2^
			MI events	2.6–3.5 m·s^−2^
			HI events	> 3.5 m·s^−2^
		AU	Player load	
		AU·min^−1^		
Ibanez et al. (2024)	Microtechnology (WIMU PRO)	m·min^−1^	Distance covered	-
			Walk	0–6 km·h^−1^
			Jog	6–12 km·h^−1^
			Run	12–18 km·h^−1^
			High intensity run	18–21 km·h^−1^
			Sprint	21–24 km·h^−1^
			Maximum sprinting	> 24 km·h^−1^
		km·h^−1^	Maximal speed	-
			Average speed	-
		n·min^−1^	Acceleration	> 0.1 m·s^−2^
		m·min^−1^	Acceleration	
		n·min^−1^	Deceleration	> −0.1 m·s^−2^
		m·min^−1^	Deceleration	
		m·s^−2^	Maximal acceleration	-
		m·s^−2^	Maximal deceleration	-
		AU·min^−1^	Player load	-
		n·min^−1^	Jumps	-
				
Gervasi et al. (2023)	TMA	meters	Distance covered	-
		% TT	Stand	0–0.7 km·h^−1^
			Walk	0.8–6 km·h^−1^
			Jog	6.1–12 km·h^−1^
			Low speed	12–15 km·h^−1^
			Moderate speed	15.1–18 km·h^−1^
			High speed	18.1–21 km·h^−1^
			Maximal speed	> 21.1 km·h^−1^
			Maximal acceleration	≥ 3.1 m·s^−2^
			High acceleration	2.1–3 m·s^−2^
			Intermediate acceleration	1.1–2 m·s^−2^
			Low acceleration	0.1–1 m·s^−2^
			Low deceleration	–0.99–0 m·s^−2^
			Intermediate deceleration	–1.99–1 m·s^−2^
			High deceleration	≤ −3–2 m·s^−2^
			Maximal deceleration	< −3 m·s^−2^
Gamonales et al. (2023)	Microtechnology (WIMU PRO)	m·min^−1^	Distance covered	-
		m·min^−1^	Explosive distance	> 1.12 m·s^−2^
		n·min^−1^	Acceleration	-
		n·min^−1^	Deceleration	-
		m·min^−1^	Distance high-speed running	> 21 km·h^−1^
		m·s^−2^	Maximal acceleration	-
		m·s^−2^	Maximal deceleration	-
		km·h^−1^	Average speed	-
		km·h^−1^	Maximal speed	-
		AU	Player load	-
		number	Jumps	.

TMA: time motion-analysis; % TT: percentage of total time; LI: low intensity; MI: moderate intensity; HI: high intensity; AU: arbitrary units; COD: change of direction

**Table 4:** Methodological approaches of studies focused on physical performance.

Study	Methods used to measure external demands	Units	Variable	Threshold
Stone et al. (2022)	Microtechnology (IMU, KINEXON Precision Technologie, 20-Hz)	AU	Total mechanical loads	
		total load	Jumps	> 0.3 s
		number	Acceleration	> 1.5 m·s^−2^
		number	Deceleration	< 1.5 m·s^−2^
		mi·h^−1^	Average speed	-
Garcia et al. (2022d)^1^	Microtechnology (WIMU PRO, 100-Hz, 10-Hz GPS)	meters	Distance covered	
		meters	Distance covered at >18 km·h^−1^	
		meters	Distance acceleration	≥ 2 m·s^−2^
		meters	Distance deceleration	≤ –2 m·s^−2^
		number	Acceleration	≥ 2 m·s^−2^
		number	Deceleration	≤ –2 m·s^−2^
Garcia et al. (2022c) ^2^	Microtechnology (WIMU PRO, 100-Hz, 10-Hz GPS)	meters	Distance covered	-
		meters	Distance covered at >18 km·h^−1^	-
		meters	Distance covered at >21 km·h^−1^	-
		number	Sprints	> 18 km·h^−1^
		number	Sprints	> 21 km·h^−1^
		number	Accelerations	> 3 m·s^−2^
		number	Decelerations	< 3 m·s^−2^
Garcia et al. (2022b)	Microtechnology (WIMU PRO, 100-Hz, 10-Hz GPS)	m·min^−1^	Distance covered	-
		m·min^−1^	High-speed running	-
		n·min^−1^	Accelerations	> 3 m·s^−2^
		n·min^−1^	Decelerations	< 3 m·s^−2^
Garcia et al. (2022a)	Microtechnology (WIMU PRO, 100-Hz, 10-Hz GPS)	meters	Distance covered	-
		meters	Distance covered at >18 km·h^−1^	-
		number	Accelerations	> 3 m·s^−2^
		number	Decelerations	< 3 m·s^-2^
Williams et al. (2021)	Microtechnology (OptimEye s5, Catapult Innovation)	AU	Player load	-
		AU·min^−1^		-
		number	HI inertial movement analysis	> 3.5 m•s^−2^
		n·min^−1^		> 3.5 m•s^−2^
		number	Inertial movement analysis	-
Saucier et al. (2021)	Microtechnology (model and sampling rate was not specified)	km	Distance covered	-
		m·s^−1^	Average speed	-
		m·s^−1^	Average maximal speed	-
		number	Jumps	-
		number	Accelerations	≥ 1.42 m·s^−2^
		number	Decelerations	≤ 1.42 m·s^−2^
		number	High accelerations	≥ 3.5 m·s^−2^
		number	High decelerations	≤ 3.5 m·s^−2^

AU: arbitrary units; HI: high intensity; ^1^ data considered peak physical demands over 60 s; ^2^ data were captured and analysed over different periods of time of most demanding scenarios (30, 60, 120, 180, and 300-s rolling averages)

Table 8 highlights the methodological approaches used to assess physiological output. Eight studies used the heart rate, reported as the mean, maximal or the percentage of maximal, to measure the physiological responses in basketball matches or training sessions (Bordon et al., 2021; Daniel et al., 2017; Dehesa et al., 2015; Gamonales et al., 2023; Garcia et al., 2022b; Puente et al., 2017; Svilar et al., 2018; Vaquera et al., 2008). One study expressed the heart rate as a sum of different intensity bands (Williams et al., 2021), and two studies used the heart rate at the lactate threshold as a percentage (Daniel et al., 2016, 2017). The 10- point Borg scale was commonly used to measure the rate of perceived exertion (López et al., 2021; Sansose et al., 2021; Svilar et al., 2018; Williams et al., 2021; Yang, 2024).

**Table 5:** Methodological approaches of studies focused on physical performance.

Study	Methods used to measure external demands	Units	Variable	Threshold
Russel et al. (2021b)^1^	Microtechnology (inertial measurement unit, Catapult T6, 100-Hz)	-	Integrated load	-
Pernigoni et al. (2021) ^2^	TMA	-	Sprint	-
		-	HI specific movements	-
		-	Jump	-
	Microtechnology (IMUs, Clearsky T6, Catapult Innovation)	AU	Player load	-
		AU·s^−1^		
		seconds		
Bordon et al. (2021)	Microtechnology (Polar Team Pro)	meters	Distance covered^3^	-
			Distance covered at 13.0–17.9 km·h^−1^	-
			Distance covered at 18.0–20.9 km·h^−1^	-
			Distance covered at 21.0–22.9 km·h^−1^	-
			Distance covered at >23 km·h^−1^	-
			Average speed	-
		number	Sprints	
Heishman et al. (2020)	Microtechnology (Catapult Sport OptimEye T6 IMU system)	AU	Player load	-
		AU·min^−1^		-
		AU	2-Demensional player load	-
			1-Demensional player load	-
			HI Inertial Movement Analysis	1.5–2.5m·s^−1^
			MI inertial Movement Analysis	2.5–3.5m·s^−1^
			LI Inertial Movement Analysis	> 3.5m·s^−1^
		number	Jump	
Vázquez-Guerrero et al. (2020)	Microtechnology (WIMU PRO, 100-Hz)	AU	Player load	-
		meters	Distance covered	-
		number	Jumps	> 5 G’s forces
			HI accelerations	> 2 m·s^−2^
			HI decelerations	< –2 m·s^−2^
Salazar et al. (2020)	Microtechnology (T6 devices, Catapult, 100 Hz)	n·min^−1^	Total forward acceleration	> 3.5 m·s^−2^
			HI acceleration	> 3.5 m·s^−2^
			Total deceleration	< 3.5 m·s^−2^
			Jumps	> 0.4 m
			HI jumps	-
			Rightward/leftward lateral	-
			HI rightward/leftward lateral movements	-

TMA: time motion-analysis; AU: arbitrary units; ^1^ the study of Russel et al. (2021b) used different systems of measuring external load: ultrawideband (UWB), local positioning system (Catapult ClearSky, Catapult Sports, Melbourne, Australia) and inertial measurement unit (Catapult T6, Catapult sports, Melbourne, Australia) which were combined with match data from an OT system (Second Spectrum, Los Angeles, United States; ^2^ the study of Pernigoni et al. (2021) combined TMA and microtechnology in the same analysis

**Table 6:** Methodological approaches of studies focused on physical performance.

Study	Methods used to measure external demands	Units	Variable	Threshold
Garcia et al. (2020)	Microtechnology (WIMU PRO, 100-Hz, 10-Hz GPS)	km·h^−1^	Peak velocity	-
		meters	Distance covered	-
		meters	Distance at >18 km·h^-1^	-
		AU	Player load	-
		number	Accelerations	> 2 m·s^−2^
		number	Decelerations	< 2 m·s^−2^
		number	Jumps	> 3 G’s forces
		number	Impacts	> 8 G’s forces
Ferioli et al. (2020)	TMA	n·min^−1^	REC	-
		% of LT	LI specific movements	-
			MI specific movements	-
			HI specific movements	-
Trapero et al. (2019)	Microtechnology (WIMU PRO)	-	Maximal accelerations	-
			Maximal decelerations	-
			Average accelerations	Jumps and impacts > 5 G’s forces
			Average deceleration	Jumps and impacts > 5 G’s forces
		n·min^−1^	Accelerations	-
			Decelerations	-
Vázquez-Gerrero et al. (2018)	Microtechnology (Triaxial accelerometer, model ADXL326, 100-Hz)	number	Moderate accelerations	< 3.0 m·s^−2^
			Moderate decelerations	< 3.0 m·s^−2^
			Maximal accelerations	> 3.0 m·s^−2^
			Maximal decelerations	> 3.0 m·s^−2^
Svilar et al. (2018)	Microtechnology (Catapult Innovations S5, 100-Hz)	number	Total forward acceleration	> 3.5 m·s^−2^
			HI acceleration	> 3.5 m·s^−2^
			Total deceleration	< 3.5 m·s^−2^
			Jumps	> 0.4 m
			HI jumps	-
			Rightward/leftward lateral	-
			HI rightward/leftward lateral movements	-
Zhang et al. (2017)	-	mi·min^−1^	Distance covered	-
			Average speed	-

TMA: time motion-analysis; AU: arbitrary units; REC: recovery

**Table 7:** Methodological approaches of studies focused on physical performance.

Study	Methods used to measure external demands	Units	Variable	Threshold
Puente et al. (2017)	Microtechnology (GPS, SPI PRO X, 15-Hz)	m·min^−1^	Distance covered	
			Stand/walk	≤ 6 km·h^−1^
			Jog	6.1–12 km·h^−1^
			Run	12.1–18 km·h^−1^
			High-speed running	18.1–24 km·h^−1^
			Maximal speed running^1^	> 24 km·h^−1^
			Sprint	> 18 km·h^−1^
			Accelerations	-
			Decelerations	-
Torres-Ronda et al. (2016)	TMA	seconds	LI specific movements	< 6 km·h^−1^
		occurrences·min^−1^ (LT)	MI specific movements	6–9 km·h^−1^
		% of LT	HI specific movements	> 9 km·h^−1^
			Stand	-
			Walk	-
			Jog/run	-
			Sprint	-
			Jump	-
			Static exertion	-
Scanlan et al. (2015)	TMA	counts·min^−1^	Stand/walk	< 3.6 km·h^−1^
		s·min^−1^	Jog	3.61–10.8 km·h^−1^
		m·min^−1^	Run	10.8–25.2km·h^−1^
			Sprint	> 25.2 km·h^−1^
			LI shuffle	defensive stance < 7.2 km·h^−1^
			HI shuffle	offensive stance > 7.2 km·h^−1^
			Dribble	-
			Jump^2^	-
			Upper body^2^	-
			Total of actions	-
Mateus et al. (2015)	-	meters	Distance covered	-
		km·h^−1^	Average speed	-
Scanlan et al. (2011)	TMA	seconds	Stand/walk	0–1.0 m·s^−2^
		meters	Jog	1.1–3.0 m·s^−2^
			Run	3.1–7.0 m·s^−2^
			Sprint	>7.0 m·s^−2^
			LI shuffle	≤ 2.0 m·s^−2^
			HI shuffle	> 2.0 m·s^−2^
			Dribble	-
			Jump	-
			Upper body	-
			Total of actions	-

TMA: time motion-analysis; % LV: percentage of live time; ^1^ maximal speed was also collected in km·h^−1^; ^2^ duration (s·min^−1^) and distances (m·min^-1^) were not obtained for jumps and upper body movements

**Table 8:** Methodological approaches of studies focused on physiological output.

Study	Methods used to measure external demands			
	HR: avg, max	HR: TRIMP, IntZon	RPE, effort intensity	RPE, session
Yang et al. (2024)				×
Gamonales et al. (2023)	×			
Garcia et al. (2022b)	×			
Williams et al. (2021)		×	×	×
Sansone et al. (2021)				×
López et al. (2021)			×	×
Bordon et al. (2021)	×			
Svilar et al. (2018)	×		×	×
Puente et al. (2017)	×			
Daniel et al. (2017)	×	×		
Daniel et al. (2016)		×		
Dehesa et al. (2015)	×			
Vaquera et al. (2008)	×			

HR: heart rate; avg: average; max: maximal; TRIMP: training impulse; IntZon: intensity zone; RPE: rate of perceived exertion

Tables 9 and 10 detail the information collected from the technical and/or tactical variables. Twelve studies retrieved the data from official websites, whereas three manuscripts collected the information on the basis of game observations. A considerable percentage of the technical and tactical papers (75%) did not report any statistical variable of data quality. Offensive and defensive technical variables were consistent across studies, but only two focused on tactical actions (Calvo et al., 2017; Courel-Ibánez et al., 2017). Three studies combined technical variables to obtain performance basketball metrics (Sansone et al., 2021; Sindik, 2015; Vázquez-Guerrero et al., 2020).

**Table 9:** Methodological approaches of studies focused on technical and tactical performance.

Study	Source of data	Data quality	Offensive variables	Defensive variables
Madinabeitia et al. (2023)	Box-score	NR	Points, successful free throws, unsuccessful free throws, successful 2-point field-goals, successful 3-point field-goals, unsuccessful 2-point field-goals, unsuccessful 3-point field-goals, dunks, fouls received, offensive rebounds, dunks, fouls received, offensive rebounds, blocks received	Fouls committed, defensive rebounds, blocks made, steals
Wang and Zheng (2021)	Box-score	NR	Successful field goal	
Sansone et al. (2021) ^1^	Game observation	NR	Points, assist, fouls received, unsuccessful field goals, unsuccessful free throws, turnovers, shots rejected	Steals, blocks, fouls committed, fouls committed
Escudero-Tena et al. (2021)	Box-score	NR	Successful 2-point field-goals, successful 3-point field-goals, successful free throws, 2-point field goals attempted, 3-point field goals attempted, free-throws attempted, offensive rebounds, assists, dunks, fouls received	Defensive rebounds, steals, blocks, fouls committed
Vázquez-Guerrero et al. (2020) ^1^	NR	NR	Points, assists, field goals attempted, free throws attempted, fouls received, missed field goals, shots rejected, missed free throws, assists, offensive rebounds	Steals, blocks, fouls committed, turnovers, defensive rebounds
Gomez et al. (2018)	Box-score	ICC = 1.0	Free-throws	
Zhang et al. (2017)	Box-score	ICC = 1.0 (free throws, two-and three-pointers, offensive and defensive rebounds, turnovers, steals, blocks, personal fouls, passes; ICC = 0.91 (assists, touches)	Successful 2-point field-goals, successful 3-point field-goals, successful free throws, 2-point field goals attempted, 3-point field goals attempted, free-throws attempted, offensive rebounds, touches, passes, assists	Defensive rebounds, steals, blocks
Courel-Ibánez et al. (2017)	Systematic observation, video analysis	Multi-rater *k* free index, Cohen’s Kappa > 0.87	Pass, reception	
Calvo et al. (2017)	Systematic observation, video analysis	NR	This study analysed mismatch situations after screening considering the effectiveness of attackers and defenders.	
Sindik (2015) ^1^	Box-score	NR	Successful 2-point field-goals, successful 3-point field-goals, successful free throws, offensive rebounds, assists, turnovers	Defensive rebounds, steals, blocks, personal fouls

NR: not reported; r: reliability coefficient; ICC: intra-class correlation coefficient

1 Variables were combined to estimate indexes of performance

**Table 10:** Methodological approaches of studies focused on technical and tactical performance.

Study	Source of data	Data quality	Offensive variables	Defensive variables
Mateus et al. (2015)	Box-score	NR	Successful 2-point field-goals, successful 3-point field-goals, successful free throws, 2-point field goals attempted, 3-point field goals attempted, free-throws attempted, offensive rebounds, touches, passes, assists	Steals, blocks, personal fouls
Sindik and Jukić (2011) ^1^	Box-score	NR	Successful 2-point field-goals, successful 3-point field-goals, successful free throws, turnovers, unsuccessful 2-point field-goals, unsuccessful 3-point field-goals, unsuccessful free throws, assists, offensive rebounds, turnovers	Defensive rebounds, fouls, steals, blocks
Escalante et al. (2010)	Box-score	NR	Successful 2-point field goals, successful 3-point field goals, successful free throws, offensive rebounds, assists, turnovers	Defensive rebounds, fouls, steals, blocks
Sampaio et al. (2008)	Box-score	r > 0.92	Assists, offensive rebounds, successful 2-point field-goals, successful 3-point field-goals, successful free throws, unsuccessful 2-point field-goals, unsuccessful 3-point field-goals, unsuccessful free throws	Blocks, defensive rebounds, fouls, steals
Page et al. (2007)	Box-score	NR	Assists, turnovers, free throws made, free throw percentage, field goals made, field goal percentage, offensive rebounds, points	Steals, defensive rebounds, fouls
Sampaio et al. (2006)	Box-scores	NR	Assists, offensive fouls, successful 2-point field-goals, successful 3-point field-goals, successful free throws, turnovers, unsuccessful 2-point field-goals, unsuccessful 3-point field-goals, unsuccessful free throws	Blocks, fouls

NR: not reported; r: reliability coefficient

### Results of the Included Studies

The information of each study (mean ± standard deviation, classification by playing position) and the variables of each outcome (physical, physiological, technical/tactical) were retrieved and combined when possible.

#### Physical Variables

**Figure 5:**
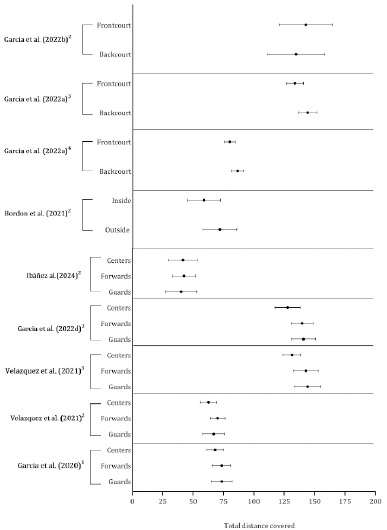
Descriptive statistics (mean ± standard deviation) of total distance covered by the playing position. ^1^ Distance covered per minute and quarter (m·min^-1.^quarter^-1^); ^2^ Distance covered per minutes (m·min^-1^); ^3^ Distance covered considering the most demanding 1-min scenario; ^4^ Distance covered considering the most demanding 30-s scenario

**Figure 6:**
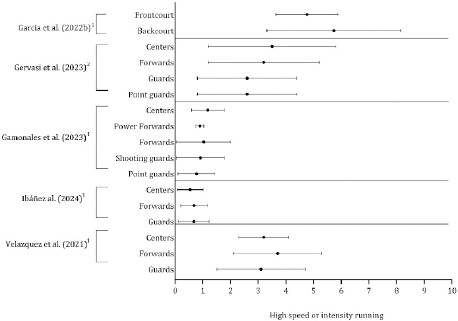
Descriptive statistics (mean ± standard deviation) of distance in high-speed running covered by the playing position. ^1^ Distance covered per minutes (m·min^-1^); ^2^ Distance covered per % of total distance

**Figure 7:**
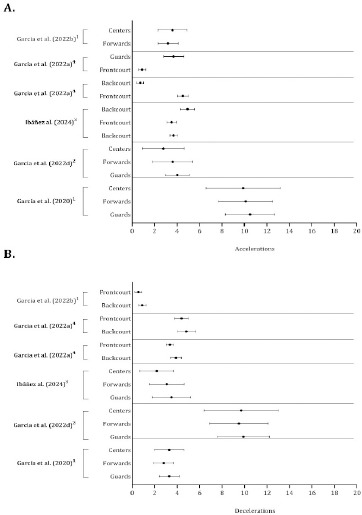
Descriptive statistics (mean ± standard deviation) of accelerations (panel A) and decelerations (panel B). ^1^ Number per minute and quarter (number·min^−1.^quarter^−1^); ^2^ Number considering the most demanding 1-min scenario; ^3^ Distance covered per minutes (m·min^−1^); ^4^ Number considering the most demanding 30-s scenarios

As shown in Tables 3–7, physical outcomes were reported across the studies using different units (absolute or relativized per time) with different thresholds. The total distance covered, high-speed and high-intensity running, acceleration and deceleration were frequently evaluated in basketball players. Consequently, studies that presented the mean and standard deviation by playing position were combined in Figures 5–7.

Although the studies used different methods of reporting the total distance covered (quarter, minutes playing, and most demanding scenarios) (Garcia et al., 2020, 2022d; Ibanez et al., 2023; Velazquez et al., 2021), the centers tended to cover less distance, on average, than forwards and guards. Outside and backcourt positions covered more distance than frontcourt and inside positions, as shown in Figure 5.

The mean and standard deviation of high-speed and high-intensity running are illustrated in Figure 6. The results varied according to the playing position classification. The relative distance, measured in meters per minute, was greater for forwards than for guards and centers during the game (Ibanez et al., 2023; Velazquez et al., 2021). Moreover, when players were grouped as point guards, shooting guards, forwards, power forwards, and centers, the relative high-speed distance (> 21 km·h^−1^) was greater in centers (1.18 m·min^−1^), whereas the lowest value was obtained for point guards (0.77 m·min^−1^) (Gamonales et al., 2023). The percentage of high speed (18–21 km·h^−1^) expressed per percentage of total distance covered was also greater in centers (3.5%) and forwards (3.2%) than in point guards and guards (2.6% in both groups). Compared with frontcourt players, backcourt players covered a greater distance at high speeds.

Accelerations and decelerations were expressed as numbers per quarter, absolute frequencies, metres per minute or frequencies, while considering the most demanding scenarios (Garcia et al., 2020, 2022a, 2022b, 2022d; Ibanez et al., 2023). Guards performed more accelerations and decelerations than forwards and centers per quarter (Garcia et al., 2020), per minute played (Ibanez et al., 2024), and when the most intense episodes were examined (Garcia et al., 2022d). Two studies investigated the absolute number of accelerations relative to the most demanding scenarios (Garcia et al., 2022a), when relativized to minutes of playing time (Garcia et al., 2022b) and when players were grouped as backcourt and frontcourt. Both studies revealed that backcourt players performed more decelerations than frontcourt players (Figure 7).

#### Physiological Variables

**Figure 8:**
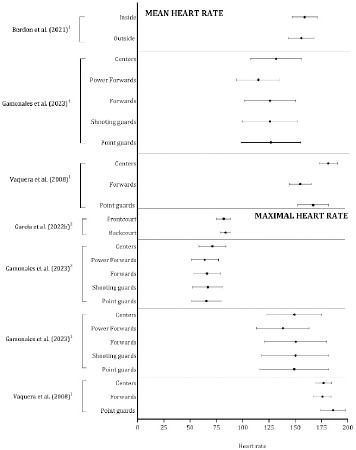
Descriptive statistics (mean ± standard deviation) of the maximal and the mean heart rate. ^1^ Studies expressed the heart rate in absolute values (beats·min^−1^); ^2^ Studies expressed the heart rate as the percentage of maximal value

As illustrated in Figure 8, independently of the data collection context (training sessions or games), the maximal heart rate (expressed in absolute values) was lower in forwards (176 ± 8 bpm) and centers (177 ± 8 bpm) than in guards (186 ± 12 bpm) (Vaquera et al., 2008). The values were lower in power forwards (138 ± 25 bpm) than in point guards (149 ± 33 bpm), shooting guards (150 ± 30 bpm), forwards (138 ± 25 bpm), and centers (149 ± 26 bpm) (Gamonales et al., 2023). The percentage of the maximal heart rate was higher in centers (71 ± 13%) than in the remaining positions (point guards: 66 ± 14%; shooting guards: 67 ± 14%; forwards: 66 ± 13%; power forwards: 64 ± 13%). In terms of the mean heart rate values, the lowest value was noted among forwards (151 ± 10 bpm) in comparison with guards (163 ± 43 bpm) and centers (177 ± 9 bpm) (Vaquera et al., 2008); additionally, the power forwards (112 ± 20 bpm) had the lowest average heart rate compared with point guards (124 ± 28 bpm), shooting guards (123 ± 25 bpm), forwards (123 ± 24 bpm), and centers (129 ± 24 bpm) (Gamonales et al., 2023). The heart rate values (i.e., percentage of maximal and mean) were comparable when the classification by playing positions used two groups: outside and inside players (Bordon et al., 2021; Garcia et al., 2022b).

**Figure 9:**
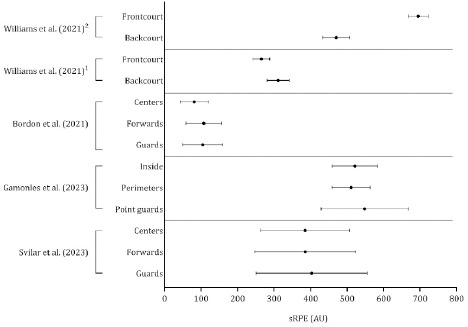
Descriptive statistics (mean ± standard deviation) of the session rating of perceived exertion. AU: arbitrary units

Data on the session rate of perceived exertion were not consistent across studies (Figure 9). An analysis of 300 training sessions (Svilar et al., 2018) and two weeks during the preseason (Gamonales et al., 2023) revealed that guards tended to assign higher values on a 10-point Borg scale than forwards and centers. In contrast, among ten professional basketball players, the weekly training load was comparable in guards (105 ± 55 AU) and forwards (107 ± 49 AU) and substantially lower in centers (81 ± 39 AU) (Bordon et al., 2021). Compared with frontcourt players, backcourt players experienced a higher session rate of perceived exertion during training sessions, whereas this trend was reversed during official games (Williams et al., 2021).

#### Technical and Tactical Variables

Game statistics of technical variables were reported in absolute values, percentages, percentages relative to minutes of playing time, and z-scores. Therefore, offensive (i.e., successful two points, successful free throws, assists, offensive rebounds) and defensive (i.e., defensive rebounds, steals) variables were commonly reported in the studies included in this review, and consequently were combined independently of the units used. As previously mentioned, only two studies investigated the tactical actions of games, and consequently, it is difficult to organize any of the results (Calvo et al., 2017; Courtel-Ibanez et al., 2017). One study concluded that the relationship between different playing positions was influenced by factors such as passing distance, ball reception, and support in defense (Courtel-Ibanez et al., 2017). Another investigation focused on tactical output and found that outside players were more accurate offensively when mismatches lasted less than five seconds (Calvo et al., 2017).

**Figure 10:**
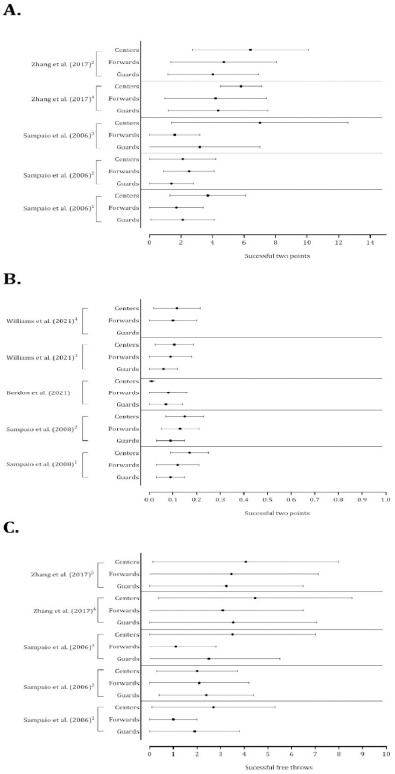
Descriptive statistics (mean ± standard deviation) of successful 2-point shots (panels A and B) and free-throws (panel C). Panel A: ^1^ Portuguese Professional Basketball League; ^2^ Spanish Professional Basketball League; ^3^ National Basketball Association; ^4^ Strong teams; ^5^Weak teams. Note: The data from Sampaio et al. (2006) and Zhang et al. (2017) were presented per minutes of playing time. Panel B: ^1^ Home teams; ^2^ Away teams; ^3^ Close games; ^4^ Balanced games; ^5^ Unbalanced games. Note: The data from Sampaio et al. (2008) and Escalante et al. (2010) were presented per minutes of playing time. Panel C: ^1^ Portuguese Professional Basketball League; ^2^ Spanish Professional Basketball League; ^3^ National Basketball Association; ^4^ Strong teams; ^5^ Weak teams. The data from Sampaio et al. (2006) and Zhang et al. (2017) were presented per minutes of playing time

**Figure 11:**
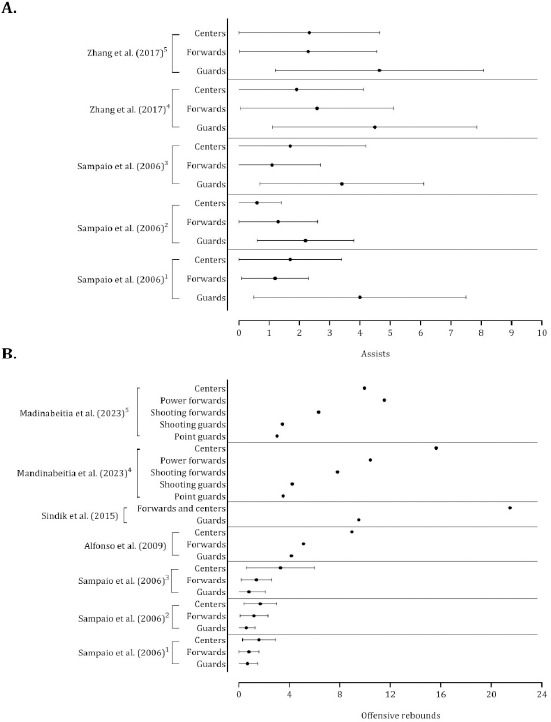
Descriptive statistics (mean ± standard deviation) of assists (panel A) and offensive rebounds (panel B). Panel A: ^1^ Portuguese Professional Basketball League; ^2^ Spanish Professional Basketball League; ^3^ National Basketball Association; ^4^ Strong teams; ^5^ Weak teams. Note: The data from Sampaio et al. (2006) and Zhang et al. (2017) were presented per minutes of playing time. Panel B: ^1^ Portuguese Professional Basketball League; ^2^ Spanish Professional Basketball League; ^3^ National Basketball Association; ^4^ National players; ^5^ Foreign players. Note: The data of Sampaio et al. (2006) and Madinabeitia et al. (2023) were presented per minutes of playing time. Alfonso et al. (2009) and Sindink et al. (2015) did not report the method to normalize data

**Figure 12:**
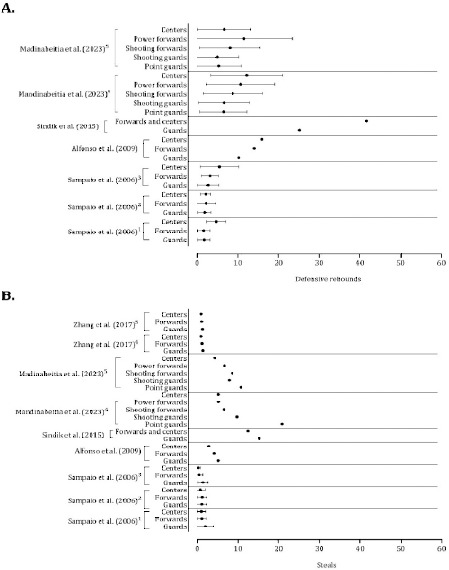
Descriptive statistics (mean ± standard deviation) of defensive rebounds (panel A) and steals (panel B). Panel A: ^1^ Portuguese Professional Basketball League; ^2^ Spanish Professional Basketball League; ^3^ National Basketball Association; ^4^ Strong teams; ^5^ Weak teams. Note: The data from Sampaio et al. (2006) and Zhang et al. (2017) were presented per minutes of playing time. Panel B: ^1^ Portuguese Professional Basketball League; ^2^ Spanish Professional Basketball League; ^3^ National Basketball Association; ^4^ Strong teams; 5 Weak teams. The data of Sampaio et al. (2006), Madinabeitia et al. (2023) and Zhang et al. (2017) were presented per minutes of playing time. Alfonso et al. (2009) and Sindink et al. (2015) did not report the method to normalize data

The accuracy of 2-point shots was systematically greater in centers and forwards than in guards (Figure 10, Panels A and B). Although substantial variability was noted, centers presented higher means of free-throw success than guards and forwards (Figure 10, Panel C). Assists discriminated among playing positions, with guards performing more assists than forwards and centers (Figure 11, Panel A). Forwards and centers tended to receive more offensive rebounds (Figure 11, Panel B). With respect to the defensive variables, centers and forwards had higher mean values of defensive rebounds (Figure 12, Panel A), and guards had higher average values of ball steals (Figure 12, Panel B).

## Discussion

This scoping review brings together a wide range of research on the physical, physiological, and technical/tactical demands placed on male basketball players across different playing positions. Substantial differences according to the playing position were noted in basketball demands. While many studies have examined these factors, there are noticeable inconsistencies in how they approach key variables, use terminology, and apply measurement techniques. These discrepancies make it difficult to develop clear, standardized guidelines for coaches and performance staff to follow. Despite these challenges, this review sheds light on important trends regarding the demands placed on different playing positions, providing valuable insights that can help inform more position-specific training and game strategies.

### Physical Demands

This systematic scoping review revealed a significant increase in the study of physical demands by playing position, with more than 75% of the studies published in the last four years. Five studies used time-motion analysis to monitor physical demands (Ferioli et al., 2020; Gervasi et al., 2023; Pernigoni et al., 2021; Scanlan et al., 2015; Torres-Ronda et al., 2016). The movements analysed in these studies were organized into two different groups: locomotion movements and basketball-specific movements. Examples of locomotion movements are standing, walking, high-speed running, and sprinting. Basketball-specific movements were defined as jumping and shuffling; however, inconsistencies were noted in the variables investigated across the studies. For example, one study combined standing and walking in the same zone (Scanlan et al., 2015), another study separated both variables and described specific thresholds (Gervasi e et al., 2023), and two studies combined different movement categories and classified them as low-specific movements (Ferioli et al., 2020; Torres-Ronda et al., 2016). Justifying the movement categories used in time-motion analysis studies is an additional problem. Three studies (Ferioli et al., 2020; Pernigoni et al., 2021; Torres-Ronda et al., 2016) mentioned a highly cited study on the topic (McInnes et al., 1995), which did not describe any rationale for the development of the eight movement categories (stand/walk, jog, run, stride, sprint, low shuffle, medium shuffle, high shuffle, jump) (McInnes et al., 1995). The remaining studies (Gervasi et al., 2023; Scanlan et al., 2015) justified the use of specific thresholds on the basis of not only basketball samples but also other team sports (Barbero-Alvarez et al., 2008; Van Gool et al., 2013). Video-technique analysis to describe the physical demands and basketball patterns also varied across studies (Ferioli et al., 2020; Gervasi et al., 2023; Pernigoni et al., 2021; Scanlan et al., 2015; Torres-Ronda et al., 2016). Nevertheless, the data obtained varied according to the software used; it should be noted that using such software is impractical for training routines and load monitoring, as it requires a specialized analyst, and, consequently, is associated with human error (Fox et al., 2017). With this in mind, more sophisticated measures of physical demands have been recently applied to monitor basketball players (i.e., local position systems (Salazar et al., 2020; Svilar et al., 2018; Williams et al., 2021; Yang, 2024) and microsensors (Garmonales et al., 2023; Garcia et al., 2022a, 2022b, 2022c, 2022d; Ibañez et al., 2024)). However, there was no consistency in identifying zones and reporting intensity threshold devices. Moreover, the use of microtechnology devices is also questionable. For example, five studies did not report a justification for the use of particular thresholds (Garmonales et al., 2023; Garcia et al., 2022a; Ibañez et al., 2024; Saucier et al., 2021; Stone al., 2021). Given the inconsistencies among the studies, the limitations of the time-motion analysis techniques, and the fact that the definition of thresholds relies on manufacturers’ instructions, comparisons of the results regarding playing positions were limited (Russell et al., 2021a).

The combination of data derived from microtechnology suggested that guards and forwards covered more distance than centers. When players were grouped into backcourt and frontcourt players, three studies showed that backcourt or outside players covered more distance in training and competition than frontcourt or inside players (Bordon et al., 2021; Garcia et al., 2022a, 2022c). The high-speed and high-intensity running mean values were greater for forwards than guards and centers. In opposition, the accelerations and decelerations tended to be greater in guards than in forwards and centers. The higher levels of high-speed running or intense activity observed in forwards can be attributed to their repeated involvement in one-on-one situations, rebounds, and ball and off-screening scenarios (Ferioli et al., 2020). The specificity of playing roles in basketball is critical, where guards require quick actions and decision-making, forwards are more focused on shooting and other related actions far and near the basket, and centers cover a wider range of group behaviours (screen on and off the ball, pivoting or shooting out of the paint). These findings provide valuable insights into the design of training sessions (Schelling et al., 2013). However, inconsistencies between the studies should be highlighted. The study of Ibanez et al. (2024) stated, “*For subsequent analysis and comparison between groups, all variables were normalized to the same unit of time (minutes)*” (p. 3). A similar description was used by Gamonales et al. (2023) to quantify the physical demands of the preseason period in elite Spanish basketball players. It is not apparent whether the normalization of physical variables considered the time when the player was actively involved in the play or only recorded when the game clock was running (i.e., the traditional definition of minutes played). In contrast, Ferioli et al. (2020) defined live time as “*game activity when the game clock was running*”. A review of male basketball players suggested that physical demands should be analysed taking into account live and total duration methods (Stojanovic et al., 2018); however, studies that have adopted both approaches are scarce. The importance of similar methodologies for determining and reporting duration is central when comparing data among studies (Tuttle et al., 2024).

### Physiological Demands

Studies that have compared the effects of the playing position on physiological demands are less extensive. Most of the data described a global description of the heart rate during training sessions or games (mean heart, maximal heart, percentage of maximal heart rate). The global maximal and mean heart rate values were systematically lower in guards and forwards than in centers. Although heart rate monitoring allows continuous evaluation of exercise intensity (Fox et al., 2017), it is affected by several factors (psychological, nutritional, and environmental) and the heart rate response is delayed during intermittent high-intensity activities which are specific for basketball (Berkelmans et al., 2018; Mancha-Triguero et al., 2019; Russell et al., 2021a), which may lead to an underestimation of exercise intensity. As a result, the heart rate should not be used exclusively to monitor physiological demands; instead, it should be combined with other physical or physiological outcomes (Garcia et al., 2022b; Lima-Alves et al., 2021). For example, physiological demands of basketball small-sided games (i.e., 3 vs. 3) demonstrated comparable average values for the mean heart rate (expressed as a percentage of the maximal heart rate) across different game formats, including man-to-man defense in a full court, man-to-man defense in a half court, and with a reduced shot clock. These results suggest that small-sided games are effective for developing aerobic performance. On the other hand, small-sided games differ in the time spent in high acceleration zones, sprints, and jumps, indicating that these formats are distinct from the activities involved in formal games (Bredt et al., 2020). Therefore, assessing the physical and physiological demands of basketball is essential for managing training loads and addressing various aspects of basketball performance (Scanlan et al., 2014). Rates of perceived exertion or training load models based on heart rate values (i.e., training individual impulses) have also been investigated considering the effect of the playing position; however, the moment of the season assessed, and the periods of assessment varied considerably (Lopez et al., 2021; Svilar et al., 2018; Torres-Ronda et al., 2016).

### Technical and Tactical Demands

The technical variables differed across basketball positions. Studies included in the present review indicate that centers are the most successful position in two-point and one-point shooting and present better statistics in offensive and defensive rebounds. The guards are decisive in assisting and stealing the ball. Therefore, shooting training, particularly from the free-throw line, should be a priority for coaches, as guards typically show better steal statistics. Additionally, optimizing decision-making and passing training is essential for centers. Variations in the different technical variables should be noted, which may explain the data quality omissions. Importantly, authors assessed and reported data validity even when the data were extracted from an official platform (Zhang et al., 2017). Similarly, in terms of physical demands, the reporting and standardization of technical variables need to be clarified. An extensive number of technical variables were systematically reported across the studies. The standardization of the technical variables should also be uniform. Mateus et al. (2015) compared guards, forwards and centers on eleven technical variables, and reported the mean and standard deviation of the coefficient variability. Seventeen performance game actions were transformed into standardized z-scores (Zhang et al., 2017), adjustments for playing time were made in other studies (Escalante et al., 2010; Sampaio et al., 2006), and offensive and defensive actions were combined to define performance metrics (Daniel et al., 2016; Garcia et al., 2022b; Saucier et al., 2021). The excessive number of technical variables examined and the different types of reporting need careful revision.

### Limitations and Future Directions

The current scoping review highlights essential data and practical implications for basketball coaches, conditioning staff, and researchers; however, limitations need to be recognized when the findings of this study are interpreted. First, studies written solely in English, Portuguese or Spanish were included in the present review. Second, physical data were obtained from different technologies (video time-motion analysis, microtechnology). The definitions of movement categories, thresholds, and approaches used to relativize physical variables varied widely across the studies. Consequently, a consensus statement about which variables and thresholds should be used is central to assessing basketball demands accurately. Third, physiological demands were mainly examined solely via a global heart rate measurement, which has limitations. Future studies must combine heart rate measurements with other physiological or physical indicators. Fourth, investigations of tactical performance considering the role of the playing position were limited, and the relative values of technical variables also differed considerably across studies; therefore, comparing studies requires caution. Recently, it was recommended that players be grouped into two categories, backcourt and frontcourt (Russell et al., 2021a), but the current review highlights the differences within these categories. For example, centers and forwards differ significantly in terms of physical, physiological, and technical/tactical demands, highlighting the limitations of categorising players into only two positional groups. Additionally, few studies have analysed the demands of basketball during training sessions, making it difficult to separate data from training and match contexts. Moreover, more than 50% of the studies reviewed focused on a single team, resulting in a limited sample size of players, games, and training sessions. This small sample size could impact the validity of the conclusions drawn. Future research should involve multiple teams over the course of an entire season to provide more robust findings when comparing the physical, physiological, and technical/tactical demands of different playing positions.

## Conclusions

Despite the considerable number of publications on male professional and semiprofessional basketball players, consistency in the methods used to monitor the physical, physiological, technical, and tactical demands is necessary to draw unequivocal conclusions. However, combining different metrics independent of data relativization revealed that guards covered more distance than forwards and centers, and performed more accelerations and decelerations. Relative high-speed or high-intensity running was higher in forwards. Physiological demands, expressed as a global description of the heart rate, indicated higher relative values for centers than for guards and forwards. Although the variation in the technical data was noticeable, the accuracy of two points, free throws, and rebounds gained, discriminated centers against guards and forwards. Given that centers and forwards differ significantly in terms of physical, physiological, and technical variables, it is not advisable to group both positions together as frontcourt players. Therefore, when interpreting these variables, at least three distinct playing positions should be considered. The present review focuses on the variability of playing positions, considers different basketball demands, and provides new insights for practitioners and researchers. Coaches and conditioning staff should understand that examining the physical, physiological, and technical variables needs to consider the position on the court. Researchers should develop a consensus statement to standardize playing position categories, variables of interest, and methodological procedures.
